# Comparison of Digital Planimetry and Ruler Methods for the Measurement of Extraction Socket Wounds

**DOI:** 10.3390/medicina59010135

**Published:** 2023-01-10

**Authors:** Weal I. Ibraheem, Ashok Kumar Bhati, Nazeeha Ahmed Hakami, Abdulsalam Dhafer Alshehri, Mohammed Hassan M. Wadani, Fai Mohammed Essa Ageeli

**Affiliations:** 1 Department of Preventive Dental Sciences, College of Dentistry, Jazan University, Jazan 45142, Saudi Arabia; 2 Private Practitioner, Jazan 82715, Saudi Arabia; 3 Private Practitioner, Abha 61412, Saudi Arabia; 4 Dental Intern, College of Dentistry, Jazan University, Jazan 45142, Saudi Arabia

**Keywords:** wound healing, soft tissue injuries, confidence interval, software, ruler method, digital planimetry

## Abstract

*Background and objectives:* The purpose of the study was to evaluate and compare ruler and digital planimetry methods to measure extraction socket wounds. *Materials and Methods:* In total, 41 extraction socket wounds were selected for assessment of wound area by ruler and digital planimetry methods. In the simple ruler method, the periodontal probe was utilized to measure the length and breadth of the extraction wound, whereas in the digital planimetry technique, Pictzar software was used. Data were analyzed using R software version 4.1.1 and Excel. For intergroup comparisons of wound surface area, Welch *t*-tests were used, and paired *t*-tests were used for intragroup comparisons. Intra-class correlation coefficients (ICC) and 95% confidence intervals (CIs) were used to evaluate the inter-method reliabilities of surface area. *Results:* Both ruler and digital planimetry techniques showed post-operative reductions in surface area. A significant difference was reported between the two techniques; however, the ruler method measurements were overestimated compared to those obtained with digital planimetry. *Conclusions:* This study concludes that digital planimetry techniques provide more accurate results when compared with the simple ruler method.

## 1. Introduction

Wound healing is a complex process. In both monitoring the wound healing process and evaluating the success of the treatment protocol, precise measurements of wound size are important. The size and location of a wound are good predictors of the treatment outcome [1,2]. In the precise clinical management of the wound healing process and the development of new treatment measurements for a group of people, the agreement, accuracy, dependability, and feasibility of wound assessment techniques are essential considerations. Various methods for objectively measuring wound size are available, ranging from simple to complex techniques. Simple methods are commonly used, but it is important to know good measurement techniques that can help clinicians in making treatment decisions [3,4,5]. In recent decades, the primary focus has been on two-dimensional wound area measurement techniques, which can be divided into contact methods (e.g., digital and manual planimetry) and non-contact methods (e.g., the mathematical elliptical method, simple ruler method, stereo-photogrammetry (SPG), and digital imaging) [6].

In the clinical context, a variety of wound measuring techniques are utilized. These measurements need a variety of instruments, ranging from simple tools, such as a ruler, to more complicated and costly machines. The most advanced approach for measuring wound areas is using a planimetric tablet, such as the Visitrak device, or smartphone software [7,8,9]. Two laser beams can be used for linear dimension calibration, and skin curvature adjustment is the more specialized and accurate mechanism for measuring wounds [10]. The most comprehensive 3D imaging systems enable a more detailed examination of wound shape, but they are not widely used in clinical practice due to high prices and time-consuming tests [8,11]. The most common method to determine wound areas is using a ruler. By multiplying the greatest length by the greatest perpendicular width, clinicians can calculate the wound surface area [12]. This measurement is simple, quick, and affordable, but it is only mathematically accurate for square or rectangular wounds. As a result, when wound shapes differ from regular square or rectangular shapes, this results in overestimation of the wound area. The ruler approach has been proven to overestimate wound area by 10% to 44%, and accuracy decreases as the wound size increases [10,11].

Digital planimetry is another common method for calculating wound surface area. This method is available both manually and digitally. In manual form, a clear film is placed over the wound, and the manual procedure marks the boundaries. The traced film is then set on a metric grid and the wound area is estimated by counting the number of grid squares that the traced area covers. In digital planimetry, images of the wound are taken and sent to the computer. The wound margin is traced on-screen with a pointing device and the wound surface area is calculated by the software. According to extant research, digital planimetric measures are more precise and trustworthy for approximating the surface area of a wound [13]. Research comparing ruler and digital planimetry techniques has mainly been performed for the chronic lesions of the skin.

There is a lack of research focusing on extraction socket wounds using the digital planimetry method in comparison to the ruler method to assess extraction wound areas. Therefore, the aim of this study was to evaluate and compare the two techniques for assessing extraction socket wounds by measuring the surface area of the wounds immediately after extraction and one-week post-extraction.

## 2. Materials and Methods

### 2.1. Ethical Approval

The study was approved by the Scientific Research Unit of College of Dentistry, Jazan University, Saudi Arabia (CODJU-20181). Informed consent was obtained from all the participants before the commencement of the study.

### 2.2. Study Population

A convenience sampling technique was conducted in patients with at least a single tooth extraction. In total, 41 extraction socket wounds were included, irrespective of the size, location (anterior or posterior teeth), method of extraction, or patient’s age and gender.

### 2.3. Assessment of Wound Area

The area of the socket wound was measured using two different techniques: ruler method and digital planimetry method.

#### 2.3.1. Ruler Method

**Figure 1:**
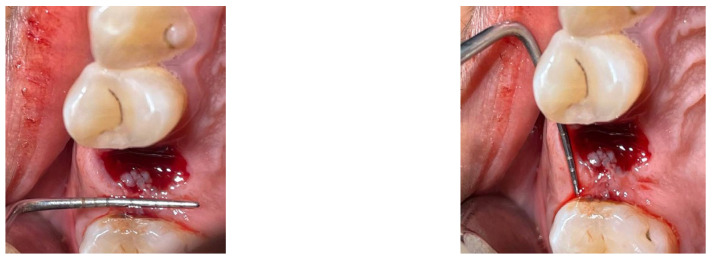
Wound measurement using the ruler method.

The basic ruler approach for calculating the surface area entails multiplying the wound’s maximum length by its maximum perpendicular breadth. It is a low-cost and easy-to-implement method. However, it is only accurate for perfectly rectangular wounds and does not account for shape variations. As a result, this approach is prone to overestimating the size of a wound. In a study conducted by Roger et al., the basic ruler approach was found to overestimate surface wounds by 41% compared with digital planimetry [13]. Similarly, Shetty et al. discovered that the basic ruler approach overestimates wound area by 29% to 43%. However, both studies used a limited sample size [14]. Periodontal probes were utilized in this study to measure the buccolingual and mesiodistal measurements of the extraction wound (Figure 1).

#### 2.3.2. Digital Planimetry Method

**Figure 2:**
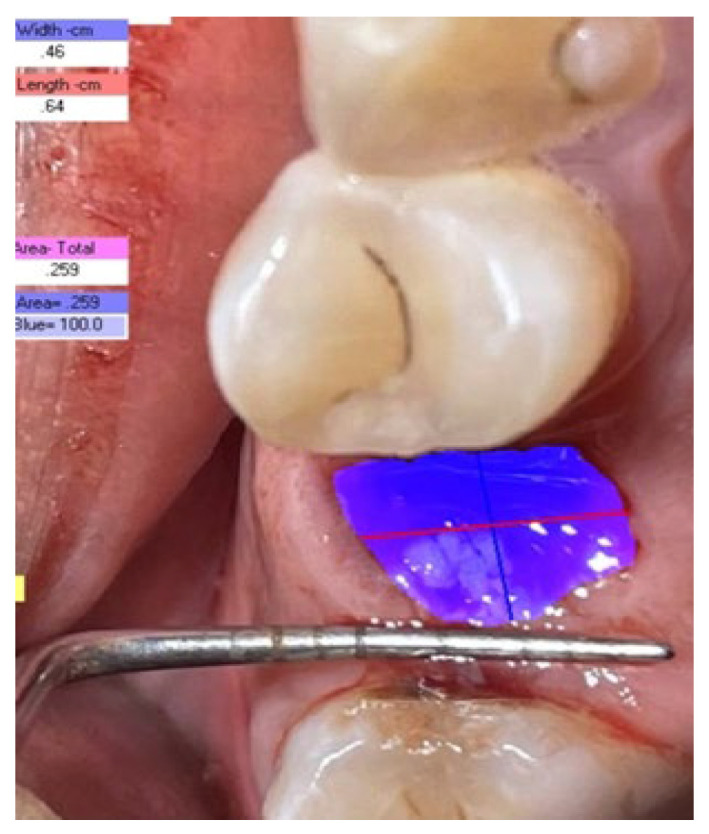
Wound measurement using digital planimetry.

Digital photographs were taken. The captured digital images were then transferred to a computer. Transferred images were analyzed to assess the wound surface area using PictZar software (Pictzar^®^-Pro version 7.6.1. SS, Elmwood Park, NJ, USA) (Figure 2). The wound margins were traced by the examiner to determine the surface area. The software determines the area by counting the pixels of the picture after it has been scaled with a ruler placed in the same plane as the lesion. The image was calibrated using a 3 mm segment of the ruler. A graduated periodontal probe was used as a ruler.

### 2.4. Data Analysis

Data were analyzed using R software version 4.1.1 and Excel. For intergroup comparisons of wound surface area, Welch *t*-tests were used, and paired *t*-tests were used for intragroup comparisons. Intra-class correlation coefficients (ICCs) and 95% confidence intervals (CIs) were used to evaluate the inter-method reliabilities of surface area. ICC values less than 0.5 are indicative of poor reliability; values between 0.5 and 0.75 indicate moderate reliability; values between 0.75 and 0.9 indicate good reliability; and values greater than 0.90 indicate excellent reliability. In order to depict the pairwise variations between ruler and digital planimetry methods, Bland and Altman plots were drawn, depicting the mean value of the pairs against the difference. These plots also demonstrate the degree of agreement between the methods. The two dotted lines on the top and bottom indicate the 95% confidence limits, within which approximately 95% of the differences between the measurements of each method should lie. The within-methods biases, in addition to their 95% upper and lower limits, were calculated.

## 3. Results

The surface areas of 41 extraction socket wounds were assessed using the standard ruler and digital planimetry techniques at baseline and at one week following extraction. Both methods revealed reductions in wound sizes at one-week post-operation. A significant difference was reported between the two techniques (Table 1).

However, it was observed that the ruler method overestimated the measurements both at baseline and one-week post-operation. When the intergroup analysis was performed, the standard ruler method had overestimated the measurements by 46.46% at baseline and 44.11% at one-week post-operation (Table 2).

### 3.1. Baseline and One-Week Post-Operative Measurements with the Ruler Method

With the ruler method, the mean surface areas of extraction wounds assessed at baseline and at one-week post-operation were 47.05 mm^2^ and 18.61 mm^2^, respectively (Table 1).

**Figure 3:**
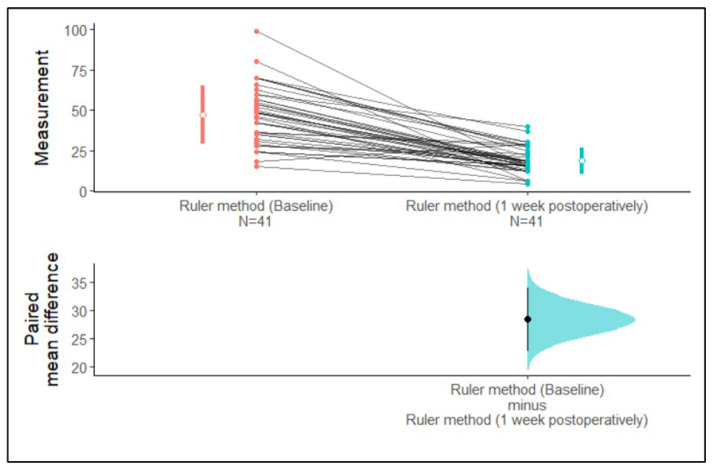
Estimation plot of the confidence intervals of the surface area assessed with the ruler method at baseline and one-week post-operation.

We observed that the 95% confidence intervals of the mean surface area of the ruler method at baseline and one-week post-operation did not overlap each other. The differences in paired mean 95% confidence intervals did not cross zero. Hence, the difference between means is greater than zero. Therefore, it can clearly be determined from Figure 3 that there is a difference in the mean surface area assessed by the ruler method between baseline and one-week post-operation (Figure 3).

### 3.2. Baseline and One-Week Post-Operative Measurements with the Digital Planimetry Method

Using digital planimetry, the mean surface areas of extraction wounds assessed at baseline and one-week post-operation were 27.74 mm^2^ and 10.40 mm^2^, respectively (Table 1).

**Figure 4:**
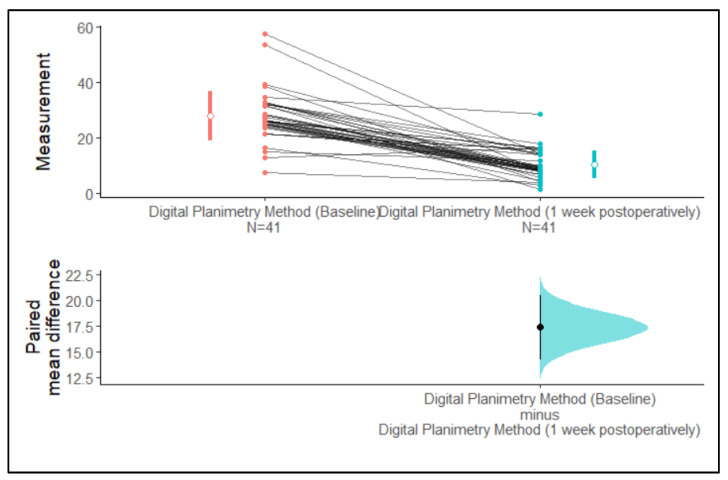
Estimation plot of the confidence intervals of the surface area assessed by the digital planimetry method at baseline and one-week post-operation.

We observed that the 95% confidence intervals of the mean surface area assessed using the digital planimetry method at baseline and one-week post-operation did not overlap with each other. The 95% confidence intervals of the paired mean difference did not cross zero. Hence, the difference between means is greater than zero. Therefore, it can be inferred from Figure 4 that there is a difference in the mean surface area assessed by the digital planimetry method between the baseline and one-week post-operative measurements (Figure 4).

**Figure 5:**
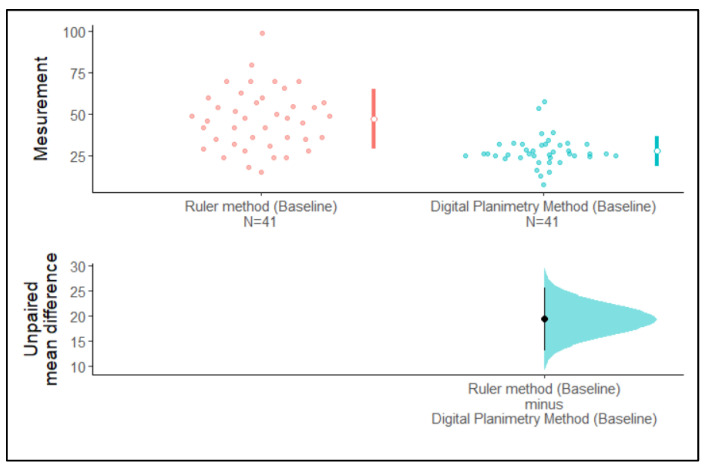
Estimation plot of the confidence intervals of surface area assessed using the ruler method and the digital planimetry method at baseline.

**Figure 6:**
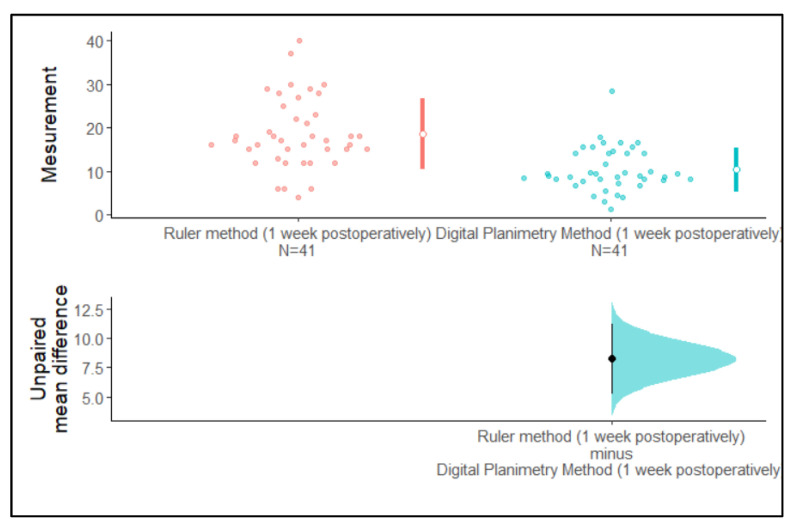
Estimation plot of the confidence intervals of surface area assessed using the ruler method and the digital planimetry method at one-week post-operation.

We observed that the 95% confidence intervals of the mean surface area assessed using the ruler method and the digital planimetry method at baseline and one-week post-operation did not overlap with each other. The 95% confidence interval of the unpaired mean difference did not cross zero. Hence, the difference between means is greater than zero. Therefore, it can clearly be stated from Figure 5 that there are differences between the mean surface area assessed with the ruler method and the digital planimetry method at baseline and at one-week post-operation (Figure 5 and Figure 6).

**Figure 7:**
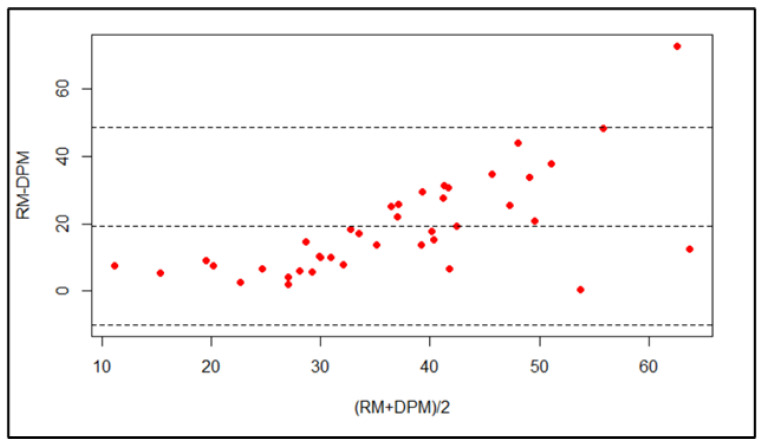
Bland–Altman plot of the surface area assessed using the ruler method and the digital planimetry method at baseline.

**Figure 8:**
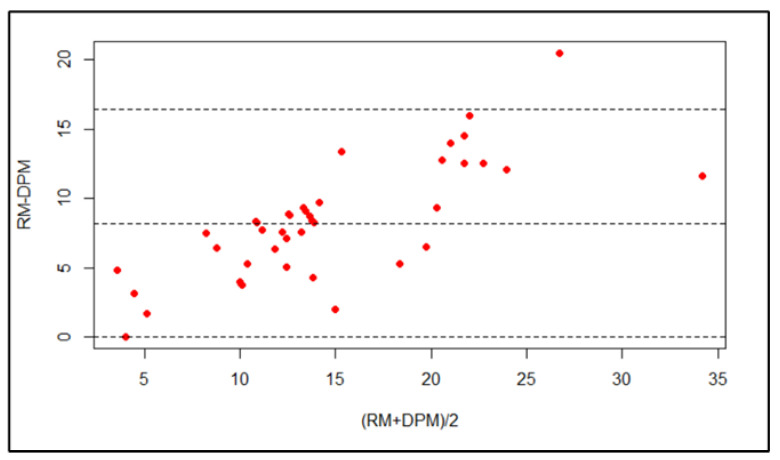
Bland–Altman plot of the surface area assessed using the ruler method and the digital planimetry method at one-week post-operation.

The Bland–Altman plot indicates that the majority of points lie within the 95% confidence interval. Hence, there is good agreement between the surface area assessed using the ruler method and the digital planimetry method at one-week post-operation (Figure 7 and Figure 8); the bias is 8.21 (95% CI: 6.89–9.53).

The lower LOA is 0.013 (95% CI: −2.26–2.29) and the upper LOA is 16.42 (95% CI: 14.14–18.69).

There was no significant interclass correlation coefficient between the ruler method and the digital planimetry method at baseline and one-week post-operation (Table 3).

## 4. Discussion

Accurate wound assessment is an essential stage in deciding appropriate treatment regimens. The continuous monitoring of wound size is important. Wound assessment techniques must be precise, dependable, and practical for use in clinical practice and clinical research investigations. Delineation of the wound border, which is sometimes difficult to identify, is one of the elements impacting the accuracy of wound measurement. Slight motions can change the appearance of a wound. The assessment methods should be able to perform consistent and accurate measurements for different types of lesions across broad geographic and clinical settings (15). Traditionally, wound-measuring methodologies have been based on two-dimensional approaches. The basic ruler method overestimates wound size if the shape deviates from square or rectangular, making it inefficient in assessing large wounds with irregular borders; however, it is quick, easy, and inexpensive [13,14,15].

Few studies have reported the non-significant and significant difference in the measured wound areas using digital and mobile cameras [8,16]. A study performed by Foltynski et al. in 2015 reported an insignificant influence of camera type on the precision of the area measured using digital planimetry and the ruler method [17].

To understand whether there were any significant or non-significant differences in values obtained using the ruler and digital planimetry methods, we calculated the *p*-values. A *p*-value of >0.05 from the simple ruler and digital methods indicated a statistically significant difference. The simple ruler technique overestimated measurements both at baseline and one-week post-extraction. Thus, it could be interpreted that a simple ruler technique, even though it is easy to learn, use, and is cost-effective, does not provide an accurate result compared with digital planimetry. The ruler approach has been proven to overestimate wound area by 10% to 44%, and accuracy decreases as the wound size increases [18,19]. Our study suggests that the simple ruler technique overestimates the wound size by 44% to 46%. Similar findings were reported by Oien et al., who compared 4 wound measurement techniques in 50 leg ulcers [20]. The conclusion drawn by these researchers was that the ruler method does tend to overestimate the measurement. Similarly, Cutler et al. evaluated different approaches for determining ulcer size and found that the values obtained from length and breadth measurements overestimate the ulcer area compared with surface area assessed by digital planimetry [21]. However, in studies performed by Wendelken et al., it was concluded that simple ruler measurements are only reliable when a wound is of a smaller size (1–4 cm^2^) and a regular shape [22]. Thus, the traditional method of measuring wound area, even though it overestimates the results in a larger wound, can be used when the wound is small.

The variations that might have been observed while using the ruler and planimetry are easily demonstrated. Multiplying the area of an irregular wound by its length and breadth usually gives the area of a rectangle. This has the added difficulty that if part of the wound heals, the greatest length and width may not change, leading to the incorrect conclusion that the wound size has not altered. As a result, length multiplied by breadth offers a rough estimate of wound size, but it is not a precise depiction of the area [22]. Similarly, the present study reported an overestimation of the measurements by the ruler method.

Intra- and intergroup evaluations were performed between both techniques: the simple ruler method showed a considerable difference compared with the digital planimetry method. Similarly, in a study performed by Sugama et al., it was reported that digital planimetry has both a high inter- and intra-rater reliability as compared with the ruler method [23]. Digital planimetry produces faster and more accurate measurements [24]. Gethin (2006) studied 50 wound tracings of superficial leg ulcers with 25 tracings smaller than 10 cm^2^ and 25 tracings larger than 10 cm^2^; they measured wound area by acetate tracing and Visitrak digital planimetry [7]. The authors concluded that more precise measurements were seen with digital planimetry. Yang et al. (2008) assessed the wound area of diabetic cutaneous ulcer surfaces with digital planimetry and transparent acetate tracing and found that digital planimetry provides accurate results [25]. The review conducted by Khoo and Jansen also reported that digital planimetry provides more accurate results [26]. In addition, our study found that digital planimetry is more accurate and precise.

Although the results of this study showed that digital planimetry gives more accurate and precise readings in comparison to the ruler method, there are certain limitations to the study. The sample size of the study was limited for statistical analysis with a short, single post-operative duration. Furthermore, there was no evaluation of wound healing variations in large and small socket wounds. Therefore, further studies assessing wound healing with a greater sample size and with different wound sizes at different post-operative durations should be conducted.

## 5. Conclusions

Digital planimetry provides more accurate and precise results than the ruler method for measuring the area of an extraction socket wound. Clinicians should choose measurement techniques for wound assessment that provide more reliable and accurate measurements.

## Figures and Tables

**Table 1:** Inter- and intragroup comparison of the mean surface area at baseline and one-week post-operation with ruler and digital planimetry methods.

Type of Methods	Baseline	One-Week Post- Operation	*p*-Value
Ruler Method	47.05 ± 18.01	18.61 ± 8.12	<0.001 PT *
Digital Planimetry Method	27.74 ± 8.90	10.40 ± 5.03	<0.001 PT *
*p*-value	<0.001 WT *	<0.001 WT *	-

Abbreviations: WT, Welch’s *t* test; PT, paired *t* test; * indicates statistical significance.

**Table 2:** Overestimation of surface area measurements by the ruler method at baseline and one-week post-operation.

	Baseline	One-Week Post-Operation
Ruler Method ^b^	47.05	18.61
Digital Planimetry Method	27.74	10.40
Difference ^a^	19.31 *	8.21
Percentage overestimation by the ruler method ^c^	46.6%	44.11%

c = a/b * 100. * indicates statistical significance.

**Table 3:** Interclass correlation coefficient of the ruler method and the digital planimetry method at baseline and one-week post-operation.

Time Point	ICC (95% CI)	*p*-Value
Baseline	0.232 (0–0.533)	0.109
One-Week Post-Operation	0.466 (0–0.795)	0.104

Abbreviation: ICC, intraclass correlation coefficient.

## Data Availability

Data included in study.
